# Pregabalin improves axon regeneration and motor outcome in a rodent stroke model

**DOI:** 10.1093/braincomms/fcac170

**Published:** 2022-06-27

**Authors:** Christof Kugler, Nelli Blank, Hana Matuskova, Christian Thielscher, Nicole Reichenbach, Tien-Chen Lin, Frank Bradke, Gabor C Petzold

**Affiliations:** Vascular Neurology Laboratory, German Center for Neurodegenerative Diseases (DZNE), 53127 Bonn, Germany; Vascular Neurology Laboratory, German Center for Neurodegenerative Diseases (DZNE), 53127 Bonn, Germany; Vascular Neurology Laboratory, German Center for Neurodegenerative Diseases (DZNE), 53127 Bonn, Germany; Vascular Neurology Laboratory, German Center for Neurodegenerative Diseases (DZNE), 53127 Bonn, Germany; Vascular Neurology Laboratory, German Center for Neurodegenerative Diseases (DZNE), 53127 Bonn, Germany; Axon Growth and Regeneration Laboratory, German Center for Neurodegenerative Diseases (DZNE), 53127 Bonn, Germany; Axon Growth and Regeneration Laboratory, German Center for Neurodegenerative Diseases (DZNE), 53127 Bonn, Germany; Vascular Neurology Laboratory, German Center for Neurodegenerative Diseases (DZNE), 53127 Bonn, Germany; Division of Vascular Neurology, University Hospital Bonn, 53127 Bonn, Germany

**Keywords:** stroke, axon growth, astrocytes, glial scar, regeneration

## Abstract

Ischaemic stroke remains a leading cause of death and disability worldwide. Surviving neurons in the peri-infarct area are able to establish novel axonal projections to juxtalesional regions, but this regeneration is curtailed by a growth-inhibitory environment induced by cells such as reactive astrocytes in the glial scar. Here, we found that the astroglial synaptogenic cue thrombospondin-1 is upregulated in the peri-infarct area, and hence tested the effects of the anticonvulsant pregabalin, a blocker of the neuronal thrombospondin-1 receptor Alpha2delta1/2, in a mouse model of cortical stroke. Studying axonal projections after cortical stroke in mice by three-dimensional imaging of cleared whole-brain preparations, we found that pregabalin, when administered systemically for 5 weeks after stroke, augments novel peri-infarct motor cortex projections and improves skilled forelimb motor function. Thus, the promotion of axon elongation across the glial scar by pregabalin represents a promising target beyond the acute phase after stroke to improve structural and functional recovery.

## Teaser

Kugler *et al*. report that reactive astrocytes in the glial scar surrounding an ischaemic stroke upregulate the synaptogenic cue thrombospondin-1, thus curtailing novel axonal connections. Inhibiting the neuronal thrombospondin-1 receptor Alpha2delta1/2 with the clinically approved drug pregabalin augments axon elongation and peri-infarct motor cortex projections, and improves skilled motor function.

## Graphical Abstract

**Graphical Abstract:**
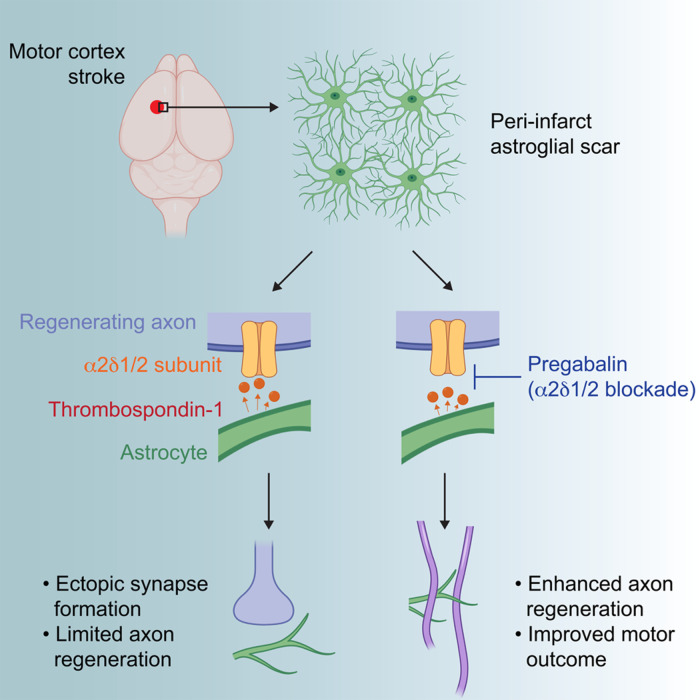

## Introduction

Ischaemic stroke therapies are limited to the first few hours after ischaemia, underlining the need for novel treatment options.^1^ One such potential therapeutic concept is the regeneration of axons of surviving neurons in the peri-infarct area.^2^ Within the first days and up to months after focal ischaemic stroke in rodents, primates and humans, these axons sprout and establish new short-range and long-range projections,^3^ resulting in plastic re-mapping of functional cortical topography.^4^ These regenerating axons, however, are restrained by a growth-inhibitory environment.^5^

In fact, the scar surrounding the lesion, which mostly contains reactive astrocytes, blocks regenerating axons. One mechanism is that reactive astrocytes secrete growth-inhibitory factors that reduce axon sprouting.^6^ However, recent studies have shown that astrocytes can also hinder axonal growth by inducing local synaptogenesis. During neuronal development, axons stop growing and switch to the formation of axodendritic or axo-glial synapses once they sense synaptogenic signals released by astrocytes.^7^ Interestingly, this endogenous switch from axon growth to synapse formation is recapitulated after CNS injury in the glial scar, where sprouting axons stop growing when they make synapse-like connections.^6,8^

A key signalling pathway underlying this switch is mediated by the α2δ2 subunit of presynaptic voltage-gated calcium channels,^9^ which is activated by the astrocyte-released synaptogenic molecule thrombospondin-1 (THBS1).^10^ Accordingly, systemic administration of the clinically approved α2δ1/2 antagonist pregabalin in a spinal cord injury model reversed this switch and re-established axonal regeneration.^9^

Here, we show that astrocyte-derived thrombospondin-1 may impede axon regeneration in the glial scar after ischaemic stroke. Specifically, we found that thrombospondin-1 is upregulated in the peri-infarct area after stroke and that pharmacological blockade of thrombospondin receptors by pregabalin improved axon sprouting and functional recovery.

## Materials and methods

### Animals

All animal research and care procedures were approved by the state’s Review Board (Landesamt für Natur, Umwelt und Verbraucherschutz of North Rhine-Westphalia, Germany) and monitored by the responsible veterinary office. All experiments were performed in accordance with institutional guidelines and the ARRIVE criteria. C57BL/6N mice (3–5 months old) were purchased from Charles River (Sulzfeld, Germany), housed in groups and monitored according to the Federation of European Laboratory Animal Science Associations recommendations under specific pathogen-free conditions. Approximately equal numbers of male and female mice were used in all experiments, and no differences were observed between sexes (data not shown). Five animals were excluded from the analysis (one mouse in the control group and four mice in the treatment group died after photothrombosis before reaching the study endpoint). Food and water were provided *ad libitum*, and mice were kept on a 12-h light/dark cycle.

### Stroke model

Mice were anesthetized with isoflurane (3% for induction, 1–1.5% during surgical procedures, v/v in O_2_), and placed in a stereotaxic frame. Body temperature was maintained at 37°C with a heating pad. As described previously,^4^ the skull over the left hemisphere was thinned with a burr drill to a circular diameter of 1 mm, with the centre of the thinned area (anterior-posterior (AP) 0 mm, medial-lateral (ML) 2 mm from bregma) positioned over the left motor forelimb area. Rose Bengal (30 mg kg^−1^ bodyweight, Sigma) was injected through the tail vein. Five minutes after the injection, an optical fibre (Doric Lenses) with an attached light-emitting diode (515 nm) was placed over the thinned skull, and the region was illuminated for 5 min to induce vascular thrombosis. Following suture, postoperative wound care and analgetic treatment with buprenorphine (0.1 mg/kg; Reckitt Benckiser), mice were placed in a warmed recovery chamber and subsequently transferred into their home cage.

### Motor behaviour assessment

As described previously,^4^ mice navigated an elevated grid structure (50 cm above ground; 30 × 30 cm; grid spacing, 1.3 cm). Videos of the mice were recorded using a digital camera (Fujifilm Finepix T350) with a mirror placed under the grid to record foot faults.

### Drug treatment

Pregabalin (Tocris) was dissolved in dimethyl sulphoxide (DMSO, Sigma), diluted in NaCl and administered intraperitoneally starting 1 h after photothrombosis (3×/day for 7 days, followed by 2×/day for 14 days) at a concentration of 46 mg kg^−1^ using a 30G syringe (B. Braun). Control animals received vehicle treatment (DMSO and NaCl without pregablin).

### Tracer injections

We used AAV9.hSyn.eGFP.WPRE.bGH virus (UPenn Vector Core) for anterograde and cholera toxin subunit B Alexa Fluor 594 conjugate (ThermoFisher) for retrograde tracing. As described,^4^ mice were anesthetized with isoflurane (3% for induction, 1–1.5% during surgical procedures, v/v in O_2_; body temperature was maintained at 37°C with a heating pad). Injection holes were drilled with a burr drill. Borosilicate capillaries (diameter, 20 mm; World Precision Instruments, WPI) attached to a syringe (10 ml, Hamilton) were mounted on a holder and lowered into the cortex. After 5 min, the tracers were injected at a rate of 100 nl min^−1^ using a pump (Ultra Micro Pump UMP3 connected to a Micro4 MicroSyringe Pump controller, WPI). Adeno-associated virus (AAV) was injected stereotactically at AP 0.5, ML 1.75, dorsal-ventral (DV) 0.5; cholera toxin B (CTb) was injected at AP 2.5, ML 1.5, DV 0.75 (all coordinates in mm from bregma). The capillary was kept in place for an additional 5 min after the injection, injection holes were sealed with bone wax (Fine Science Tools), and the skin was sutured and postoperative wound care and analgesia. Animals were allowed to recover in a temperature-controlled chamber before being transferred to their home cages.

### Whole-brain clearing

Whole-brain clearing was based on established protocols.^4^ Briefly, we washed the fixed mouse brains three times for 5 min in Milli-Q H_2_O at room temperature. Each brain was transferred to a glass vial (20 ml, Carl Roth) filled with 30% (v/v) tert-butanol (Carl Roth) solution in Milli-Q at 37°C. We used 30% tert-butanol (pH 9.9), 50% tert-butanol (pH 9.7), 70% tert-butanol (pH 9.5), 80% tert-butanol (pH 9.6), 96% tert-butanol (pH 9.5), 100% tert-butanol (pH 9.4) and BABB [1:2 mixture of benzyl alcohol (Carl Roth) and benzyl benzoate (Carl Roth), pH 9.4] for dehydration and clearing [all solutions were adjusted for pH with triethylamine (Sigma)]. Brains were incubated in each dehydration and clearing solution for 60 min at 37°C, and subsequently rendered transparent in BABB (24 h, 37°C) to. Cleared brains were stored protected from light at 4°C.

### Immunohistochemistry

Mice were anesthetized with 3% isoflurane in oxygen (v/v) and an intaperitoneal bolus of 1 mg kg^−1^ ketamine. Brains were fixed by perfusion with 15 ml ice-cold 4% (w/v) paraformaldehyde solution and stored overnight at 4°C. Fixed mouse brains were cryo-protected in 15 and 25% (w/v) sucrose solutions [in 1× phosphate-buffered saline (PBS) supplemented with 0.1% (w/v) NaN_3_] for 1 day each at 4°C. Twenty-micrometre transverse sections were cut with a cryostat (Leica) and stored in 1× PBS supplemented with 0.1% NaN_3_ at 4°C. Sections were blocked [1× PBS supplemented with 10% (v/v) normal serum and 0.5% (v/v) Triton X-100] at room temperature for 1 h, and incubated in primary antibody solutions [1× PBS supplemented with 5% (v/v) normal serum and 0.05% (v/v) Triton X-100] overnight at 4°C on a rotating platform (glial fibrillary acidic protein, GFAP: 1:250, Z0334, Dako; TSP1: 1:100, #14-9756-82, ThermoFisher; Bassoon: 1:100, ADI-VAM-PS003, Enzo; Homer1: 1:250, 160006, Synaptic Systems; neurofilament M: 1:500, 822701, BioLegend; NG2: 1:50, AB5320, Merck). After 24 h, sections were incubated at room temperature for 3 h with Alexa-conjugated secondary antibodies (ThermoFisher; Alexa Fluor-488 goat anti-rabbit IgG; Alexa Fluor-594 goat anti-mouse IgG; Alexa Fluor-647 goat anti-chicken; Alexa Fluor-488 goat anti-mouse) in 1× PBS. Sections were mounted on a glass microscopy slide with Fluoromount-G medium (supplemented with DAPI; Invitrogen) and stored protected from light.

### Confocal and two-photon microscopy

For confocal microscopy, slides were imaged using an upright Zeiss LSM700 microscope (20×/0.8 or 63×/1.4 objectives, Zeiss) and saved as 8 bit images. The same image acquisition and filter settings were used for each staining.

Confocal imaging of RNAscope probes was performed by imaging 3 µm *Z*-stacks (interval, 0.19 µm; *n* = 10 for each animal) in the glial scar or in the corresponding region in control mice.

Two-photon microscopy of cleared brains was performed as described^4^ using a Zeiss LSM 7MP upright microscope (10× objective, Olympus, XLPLN10XSVMP; NA, 0.6; working distance, 8 mm). Briefly, cleared brains were transferred to a mirrored cylinder, immersed in clearing solution and placed in a dish (diameter, 10 cm). An imaging dish (m-dish 50 mm, low; Ibidi) was placed on top of the cylinder and filled with 2,20-Thiodiethanol (Sigma). Enhanced green fluorescent protein (eGFP) was detected at 920 nm, and Alexa Fluor 594 was detected at 780 nm. Image tile scans and *Z*-stacks were acquired and saved as 8 bit images.

### RNAscope *in situ* hybridization

Multifluorescent RNAscope *in situ* hybridization (Advanced Cell Diagnostics, #320850) was performed using probes for *Sox9* and *Thsp1* (Mm-Sox9-C3, #563571-C3; Mm-Thbs1-C1, #457891) according to the manufacturer’s instructions. Briefly, mice were transcardially perfused with 20 ml ice-cold sterile 0.9% NaCl 14 days after photothrombosis or sham procedure. The brain was removed and embedded in embedding medium (OCT; Fisher Scientific, #12678646) and snap frozen with 2-methylbutane on dry ice. Frozen brains were stored at −80°C until sectioning at −20°C using a Cryostat (CryoStar NX70, Thermo Fisher Scientific). Twenty-micrometre coronal brain sections were transferred to coated glass slides (Superfrost plus, Thermo Fisher Scientific, #10149870) and stored at −80°C until usage. Sections were fixed with 4% formaldehyde for 20 min on ice and washed twice with PBS for 5 min at room temperature. The amplification steps were performed according to the manufacturer’s instructions (Amp 4 Alt-C colour option was used to label the C1 probe with Atto 550 and the C3 probe with Atto 488).

### Imaging and behavioural data analysis

For RNAscope data analysis, the number of *Thbs1*-positive astrocytes was quantified in maximum intensity projections using CellProfiler software (version 3.1.5). First, Dapi-positive nuclei were defined as objects with a diameter of 30–120 px, followed by an object expansion with 40 px to define the cell cytosol. Positive probe signals within these expanded objects were defined as dot-like structures with a diameter of 3–10 px. A cell was defined as an astrocyte if it contained >15 *Sox9* signals/cell, and as *Thbs1*-positive if it contained >5 *Thbs1* signals/cell. In each animal, 10 fields of view in two sections were analysed.

For motor behaviour analysis of the elevated grid walk test, steps from each paw were counted individually (forepaws, 50 steps; hindpaws, 30 steps) and the number of missteps was used to calculate a paw misplacement ratio for left and right forepaws and hindpaws.

All immunohistochemical data points represent mean values of 5–10 brain sections per mouse. For synaptic punctae analysis, images were imported into Fiji and binarized using an automated Otsu algorithm. Colocalized Bassoon-positive or Homer1-positive punctuate signals were counted in thresholded confocal 3D image volumes using the Synapse Counter plugin for Fiji^11^ with a predefined puncta size range of 0.9–20 μm^2^. The glial scar was defined by an area of increased GFAP intensity, measured by binarizing GFAP stainings using thresholds higher than the average baseline GFAP intensity in remote areas plus the triplicate of the standard deviation. Axon density in the glial scar was analysed by quantifying binarized anti-neurofilament M positivity in the GFAP-positive scar area using Fiji. TSP1 area coverage was quantified in binarized images using Fiji.

For quantification and analysis of neuronal CTb-positive somata, 600-µm *Z*-stacks were acquired. We imaged the injection site as well as regions anterior, medial and lateral from the infarct, and corresponding regions in control mice, respectively. Positive cells were counted using Fiji after background subtraction, smoothing using a 3D Gaussian blur filter (*xy* sigma, 5.5; *z* sigma, 2.0), greyscale conversion and segmentation using a watershed algorithm as described.^4^

Axonal projection profiles were analysed as described.^4^ Briefly, images were stitched in Fiji and subjected to the learning segmentation toolkit ilastik to binarize images according to EGFP signal or background. Individual images were registered and verified for consistency of the injection site by delineating the area of saturated EGFP signal and defining its centre as the injection site. Images were then registered based on anatomical landmarks: to register image stacks of the M1–premotor cortex (PMC) region, a perpendicular line was drawn from the brain midline to the centre of the injection site, and images were excluded from the analysis if the length of this line exceeded 1350 mm ± 10% (based on the AAV injection 1750 mm lateral from midline corrected for 23% isometric tissue shrinkage due to the clearing method); to register stacks of the M1-S1 region, points were drawn at the frontal and caudal end of the lateral brain curvature at *Z*-distances of 50, 150 and 250 mm from surface, respectively, and these points in each plane were connected by a line. Subsequently, another line perpendicular to this line was drawn from the centre of the injection site, and images were excluded from the analysis if the length of this line exceeded 1250, 1525 or 1915mm ± 10% at the three imaging depths. In the *Z*-direction, stacked images were registered from the cortical surface (*z* = 0 mm) to a depth of 600 mm. Finally, all maximal projection images from each animal were overlaid in each group, and animals were excluded if the size of the injection site exceeded ±10% from the mean.

All ilastik-processed images were registered in Fiji to create a single representation of all axonal projections, and separated into regions of interest (ROIs) I–IV. Individual ROIs were loaded in Imaris (Oxford Instruments), and axonal projection volumes (expressed in percent distribution) were calculated with the built-in surface creation wizard using thresholds higher than the average background intensity plus the triplicate of the standard deviation. These files were loaded into Imaris to visualize projections. Infarct volumes were quantified using *Z*-stack images of the infarct cavity that were analysed using the built-in surface creation wizard in Imaris. Some schematic images were prepared using BioRender (https://biorender.com).

### RiboTag RNAseq data analysis

The differentially expressed gene list from Cx43-CreER(T)::RiboTag-loxP mice subjected to middle cerebral artery occlusion (MCAO) was derived from our published RiboTag-Seq dataset GSE103783^12^ with DESeq2, using false discovery rate < 0.01 and the corresponding adjusted *P*-value < 0.05 as the filtering criteria. After filtering, 227 downregulated genes and 436 upregulated genes (stroke versus control) were retrieved. To find over-representative pathways associated with upregulated genes in the stroke group, gene ontology (GO) enrichment was performed with clusterProfiler using the total identified 12 968 genes as the background. The *P*-value cutoff was 0.01 and the *q*-value cutoff was 0.05.

### Statistical analysis

All analyses were performed by investigators blinded to the group allocation of each dataset. The sample size was predetermined based on a statistical power of 0.8 using G*Power 3 analysis software^13^ and based on previous experience. Biological replicates for each group are reported in the figure legends. All animals were randomly assigned to experimental groups using the ‘RAND( )’ function in Microsoft Excel.

We used the unpaired *t*-test or Mann–Whitney test for comparisons between two groups, and the Kruskal–Wallis test followed by Dunn’s multiple comparisons test to compare several groups. We used the two-way repeated-measures ANOVA and Bonferroni *post hoc* test for multiple measurements in the same groups, and ordinary two-way ANOVA and Bonferroni *post hoc* test to determine responses affected by two factors. All data were analysed using GraphPad Prism 9 (GraphPad Software) and are expressed as Tukey’s box-and-whisker plots indicating the median, mean, interquartile range (IQR) and 1.5 IQR. A *P*-value of <0.05 was considered statistically significant.

### Data availability

The data that support the findings of this study are available from the corresponding author upon reasonable request.

## Results

**Figure 1:**
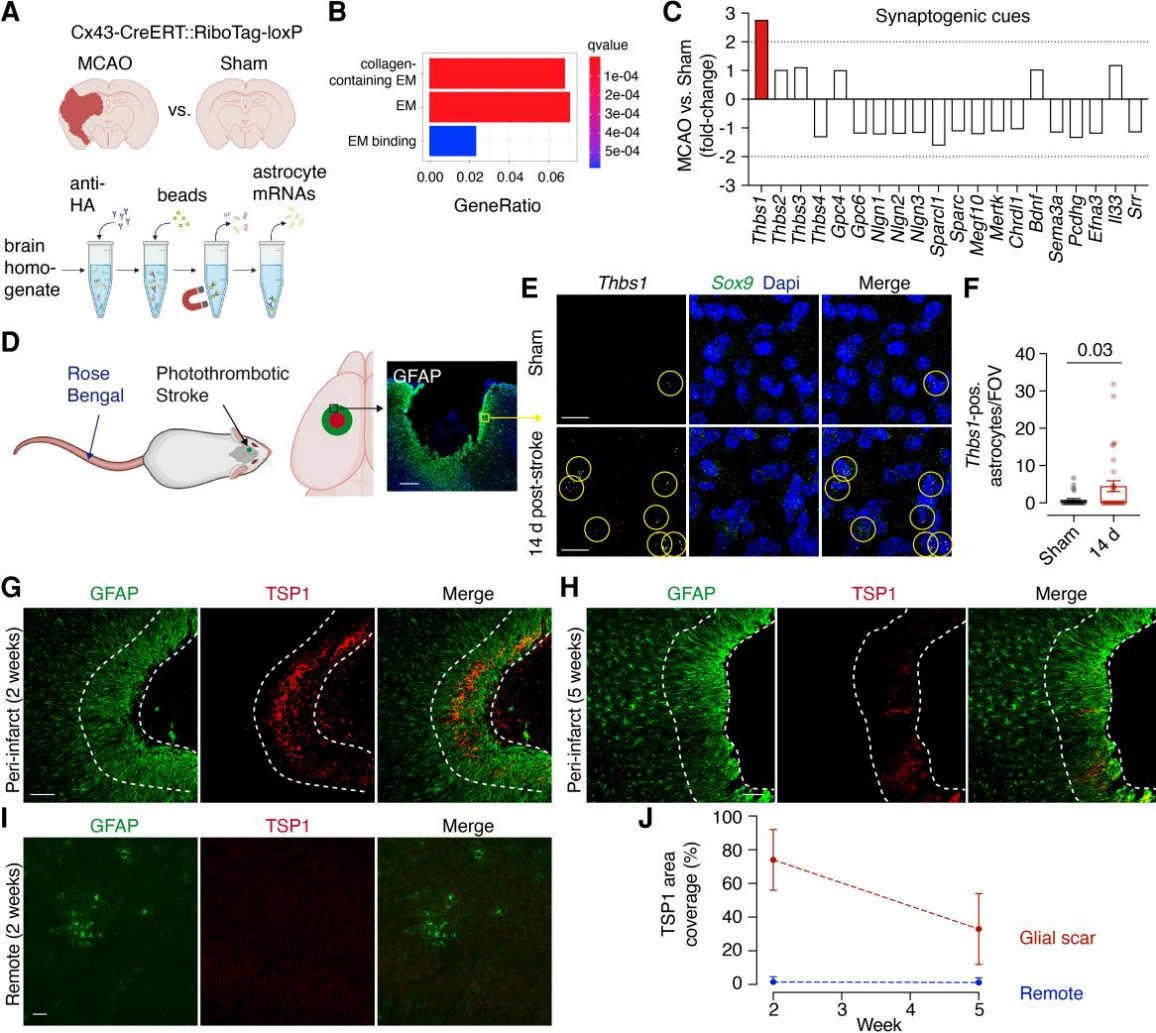
**Thrombospondin-1 expression in reactive astrocytes after stroke**. (**A**) mRNA purification workflow. Cx43-CreERT::RiboTag-loxP mice were subjected to MCAO or sham surgery. Astrocyte-specific translating mRNAs were immunopurified using anti-haemagglutinin antibodies and analysed using RNAseq. (**B**) GO analysis (EM, extracellular matrix). (**C**) Fold-change comparison from RiboTag-derived mRNA of astrocyte-expressed synaptogenic cues 72 h after stroke compared with sham. (**D**) Photothrombotic stroke induction using Rose Bengal and local irradiation shows astroglial scar formation at the infarct border (GFAP; scale bar, 300 µm). (**E** and **F**) RNAscope analysis of reactive astrocytes within the glial scar (identified by *Sox9* expression) 14 days after stroke (datapoints from *n* = 4 mice in each group; unpaired *t*-test; circles show *Thbs1*/*Sox9*/Dapi overlap; scale bar, 20 µm). (**G-I**) Immunohistochemistry for the *Thbs1*-gene product thrombospondin-1 (TSP1) 14 days and 5 weeks after stroke (the GFAP-positive glial scar is delineated by dashed lines) and in remote ipsilateral PMC 14 days after stroke (scale bars, 50 µm). (**J**) TSP1 expression levels (quantified as covered immunopositive area) in the glial scar and remote areas (PMC) 14 days and 5 weeks after stroke.

Paracrine factors released by reactive astrocytes can serve a dual role that could mediate inhibitory action to regenerating axons, in that they can act as inhibitory extracellular matrix components as well as synaptogenic cues.^14^ We, therefore, assessed changes in gene expression after stroke *in silico* focusing on extracellular matrix components. To this end, we exploited our previously published dataset reporting the gene expression profile of reactive astrocytes in mice 72 h after ischaemic stroke compared with sham surgery using the RiboTag technique^12^ (Fig. 1A). Indeed, GO analysis showed an enrichment of genes with the terms ‘extracellular matrix binding’, ‘extracellular matrix’ and ‘collagen-containing extracellular matrix’ (Fig. 1B). To further explore the role of astroglial synaptogenic cues that are part of these GO genesets, we assessed 20 astrocyte-expressed genes implicated in synapse formation or elimination.^15^ Intriguingly, we found that the gene *Thbs1*, which encodes for the α2δ1/2 ligand thrombospondin-1, showed an almost 3-fold upregulation 72 h after MCAO compared with sham, i.e. the highest increase among astrocyte-derived synaptogenic cues (Fig. 1C).

Axonal sprouting and cortical re-mapping have extensively been studied in focal cortical stroke models that result in reproducible ischaemic infarcts of the motor cortex delineated by astroglial scar tissue. Therefore, we analysed *Thbs1* expression in such a model, in which photothrombotic stroke is produced by systemic administration of a photosensitizer followed by targeted irradiation of the primary motor cortex (Fig. 1D).^4^ RNAscope *in situ* hybridization showed that the number of *Thbs1*-positive astrocytes in the peri-infarct scar area was 6-fold upregulated 14 days after stroke compared with sham (Fig. 1E and F). Immunohistochemistry confirmed that THBS1 is expressed in the astroglial scar 2 weeks and 5 weeks after stroke (Fig. 1G–I). Hence, thrombospondin-1 increases across a wide time window in two different models of acute focal stroke. However, no increase of thrombospondin-1 immunoreactivity occurred in remote ipsilateral cortical target regions of regenerating axons, such as PMC (Fig. 1I–J), implying that synaptogenic mechanisms independent of thrombospondin-1 signalling may prevail in these regions.

**Figure 2:**
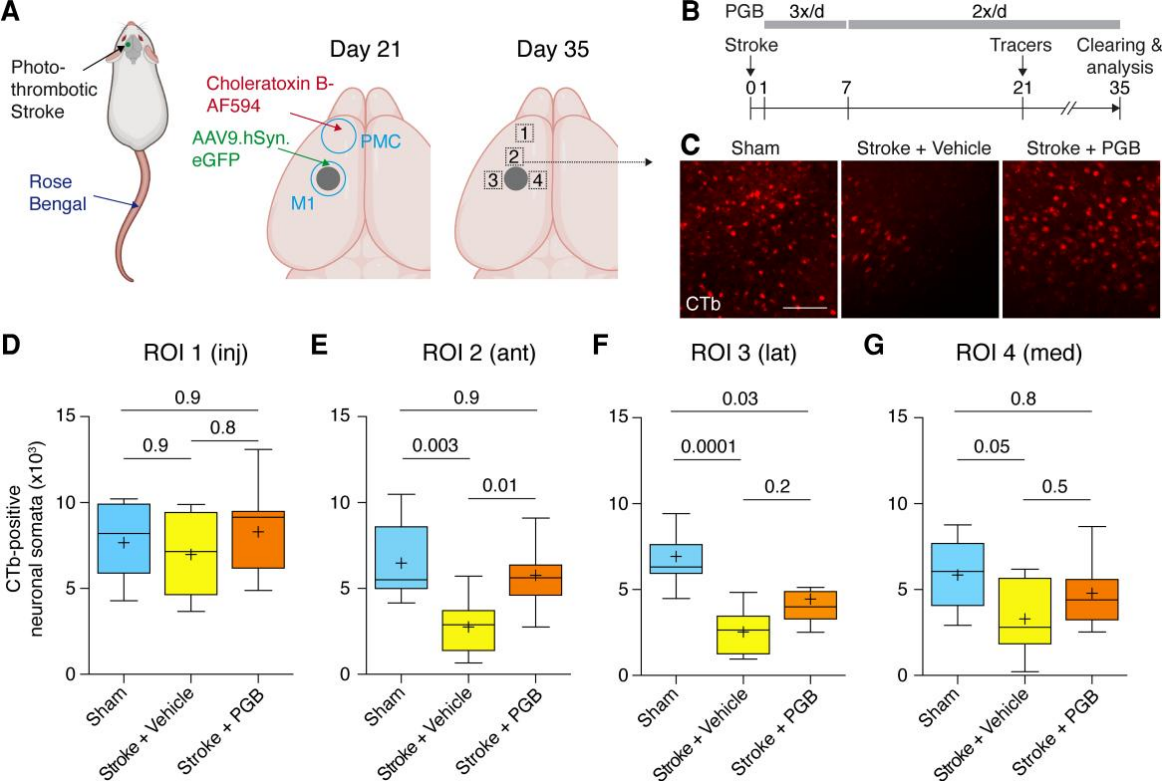
**Pregabalin improves retrograde connections after stroke**. (**A** and **B**) Experimental timeline. Mice were subjected to photothrombotic stroke and treated with pregabalin (PGB). Anterograde (AAV9.hSyn.eGFP) and retrograde (CTb) tracers were injected into motor (M1) and premotor (PMC) cortex at 21 days, and axon projections were analysed in cleared brains at 35 days. (**C**) Representative fluorescent images of CTb-positive neurons anterior to the stroke region (scale bar, 100 µm). (**D–G**) Retrograde connections were analysed in four ROIs (*n* = 10 mice in each group; Kruskal–Wallis test followed by Dunn’s multiple comparisons test).

Based on the hypothesis that pharmacological blockade of thrombospondin-1 signalling could enhance axon growth, we investigated the effects of pregabalin, an antagonist of the thrombospondin-1 receptor α2δ1/2.^16^ We induced focal cortical stroke in the forelimb area of the M1 primary motor cortex and mapped anterograde and retrograde connections after stroke (Fig. 2A) as described.^4^ In short, we treated mice systemically with pregabalin or vehicle for 5 weeks after stroke (Fig. 2B). Twenty-one days after stroke induction, we injected the fluorescently labelled retrograde tracer CTb into PMC to back-label neuronal projections from the infarcted motor cortex to the PMC. In addition, we transduced neurons in the forelimb motor cortex anterior to the infarct with AAV, encoding for an anterograde green fluorescent protein tracer, to label anterograde projections emanating from M1 (Fig. 2A–C). Two weeks later, isolated brains were rendered transparent by whole-brain organic solvent-based clearing,^4^ and anterograde and retrograde M1–PMC connections were 3D-imaged and quantitively mapped in defined areas medial, lateral and anterior to the infarct (Fig. 2A).

As expected, control mice that had undergone a sham-stroke procedure showed robust retrograde labelling from PMC to M1 (Fig. 2C–G), whereas these connections were strongly reduced after stroke in vehicle-treated mice (compared with sham: anterior, 42%; lateral, 37%; medial, 56%; Fig. 2C–G). Remarkably, pregabalin treatment approximately doubled retrograde axonal projections between M1 and PMC anterior to the infarct (209% compared with vehicle; Fig. 2C–G).

**Figure 3:**
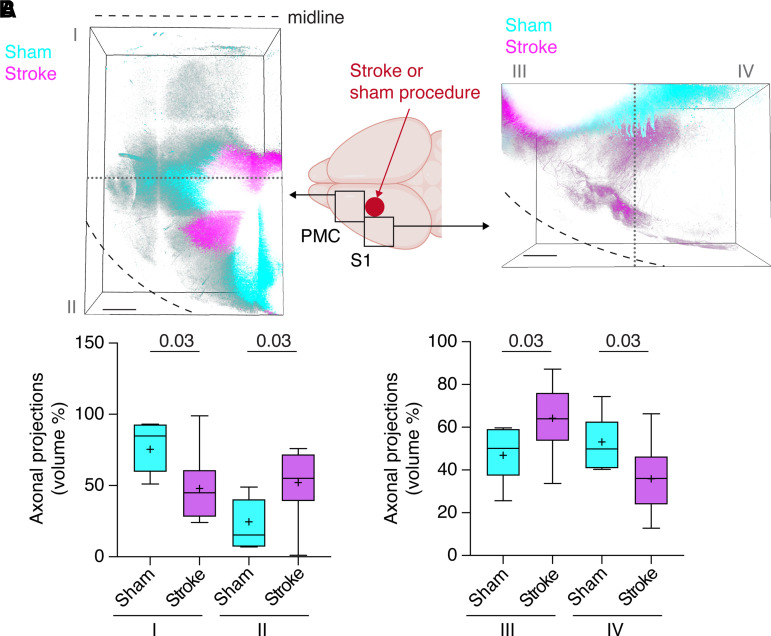
**Axonal re-mapping after ischaemic stroke**. (**A**) Anterograde EGFP labelling was assessed in PMC and somatosensory cortex (S1) anterior and medial to the infarct. These areas were divided into ROIs I–IV (summation images of all animals, depicted as colour-coded maximal intensity *Z*-projections; the grey dotted line delineates the borders between ROIs). (**B**) Compared with control mice, anterograde connections in ROIs I and IV were reduced after stroke, but increased in ROIs II and III (corresponding to anterolateral motor and somatosensory cortex; *n* = 10 stroke versus *n* = 10 sham animals, Mann–Whitney test; scale bars, 300 mm).

To confirm that axonal remodelling of anterograde connections occurs in our stroke model, we mapped fluorescently labelled anterograde axonal projections from peri-infarct cortex into PMC and M1. As reported,^4^ anterograde connections displayed a complex picture of reduced connections into anteromedial PMC and posterolateral somatosensory cortex, but increased novel connections into the anterolateral PMC and anterolateral somatosensory cortex after stroke compared with control mice reflecting axonal re-mapping (compared with sham: anteromedial PMC, 63%; anterolateral PMC, 212%; anterolateral somatosensory, 138%; posterolateral somatosensory, 67%; Fig. 3A and B).

**Figure 4:**
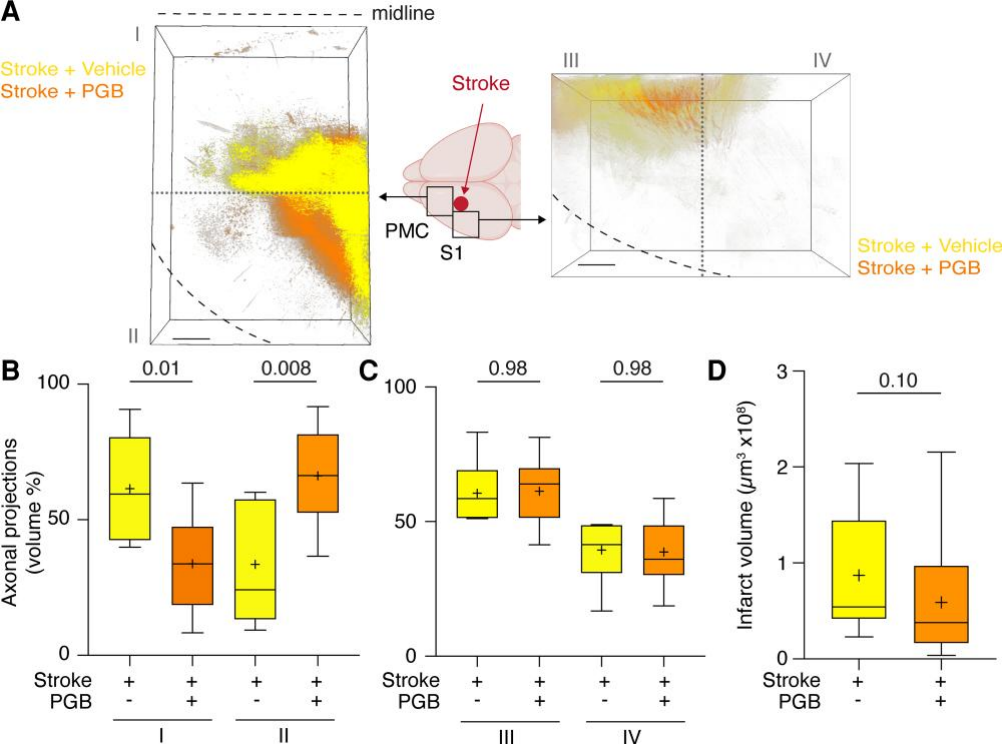
**Pregabalin improves anterograde connections and synapse formation in target regions after stroke**. (**A**) Anterograde eGFP labelling was assessed in PMC and somatosensory cortex (S1) anterior and medial to the infarct, and these areas were divided into ROIs I–IV (summation images of all animals, depicted as colour-coded maximal intensity *Z*-projections; the grey dotted line delineates the borders between ROIs). (**B** and **C**) Vehicle-treated mice after stroke were compared with mice receiving PGB treatment after stroke in ROIs I–IV (*n* = 11 mice in both groups; Mann–Whitney test; scale bars, 300 mm). (**D**) Infarct volumes were compared in both groups (*n* = 11 mice in both groups; Mann–Whitney test).

Next, we determined the effects of pregabalin. Remarkably, stroke-induced plastic cortical re-mapping of anterolateral motor connections doubled in mice treated with pregabalin compared with vehicle-treated controls (197%; Fig. 4A and B). In addition, we also observed that anteromedial motor connections, which decrease after stroke (Fig. 3A and B), were reduced further in mice treated with pregabalin (56% compared with vehicle; Fig. 4B), but that retrograde connections in that region were increased by pregabalin (Fig. 2E). No effects by pregabalin were seen on projections into anterolateral and posterolateral sensory cortex (anterolateral, 101%; posterolateral, 98%; Fig. 4C). Representative examples of brains in each group expressing anterograde tracer are shown in Supplementary Fig. 1 and Video 1 (sham), Video 2 (stroke + vehicle) and Video 3 (stroke + prebabalin). Moreover, infarct volume was similar in mice treated with pregabalin compared with vehicle-treated animals (Fig. 4D).

**Figure 5:**
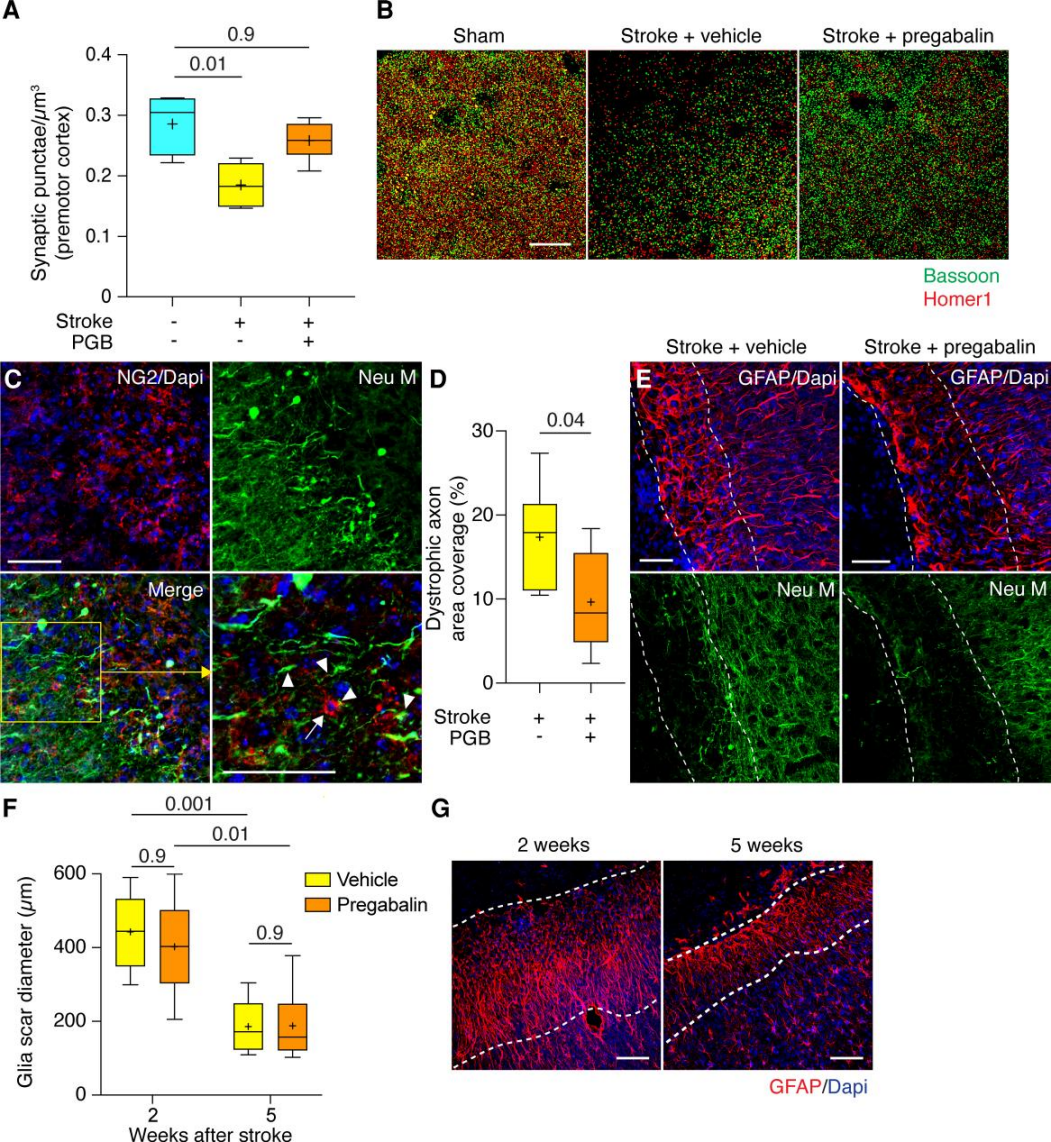
**Effects of pregabalin on axonal and synaptic density after stroke.** (**A** and **B**) Synaptic punctae in PMC were compared in confocal 3D volumes in sham-treated mice, vehicle-treated mice after stroke and pregabalin-treated mice after stroke (sham, *n* = 5 mice; stroke + vehicle, *n* = 6 mice; stroke + pregabalin, *n* = 8 mice; Kruskal–Wallis test followed by Dunn’s multiple comparisons test; images show representative examples; scale bar, 20 µm). (**C**) Dystrophic axons (anti-neurofilament M, Neu M) terminating near NG2-positive cells in the glial scar after stroke (arrowheads point to axon terminations and arrows point to NG2 cell in zoomed-in image; scale bars, 50 µm). (**D** and **E**) Dystrophic axon density (quantified as area fraction of Neu M) in the glial scar (defined by GFAP staining) in mice treated with vehicle versus pregabalin 2 weeks after stroke (stroke + vehicle, *n* = 6 mice; stroke + pregabalin, *n* = 8 mice; Mann–Whitney test; images show representative examples; scale bars, 50 µm). (**F**) Glial scar diameter 2 weeks and 5 weeks after stroke in mice treated with vehicle or pregabalin (*n* = 6 mice per group; ordinary two-way ANOVA followed by Bonferroni’s multiple comparisons test). (**G**) Representative examples (vehicle-treated mice 2 and 5 weeks after stroke; increased GFAP signal is delineated by dashed lines; scale bars, 100 µm).

To investigate if regenerating axons also form synapses in target regions, we quantified synaptic punctae—i.e. colocalization of the pre- and postsynaptic markers Bassoon and Homer1—in lateral PMC (Region II in Fig. 4A) by high-resolution confocal microscopy. As expected, the density of synaptic puncta was strongly decreased in mice 5 weeks after stroke, but similar to sham in mice treated with pregabalin after stroke (Fig. 5A and B), indicating that augmentation of axon regeneration by pregabalin is paralleled by increased synaptic circuit integration in cortical target areas. Moreover, we also quantified the density of axons ending in the glial scar. Interestingly, we observed that many axons terminated near NG2 glial cells as previously reported^8^ (Fig 5C). Moreover, the density of axons terminating in the glial scar was lower in mice treated with pregabalin compared with vehicle after stroke (Fig. 5D and E). We also analysed the diameter of the GFAP-positive glial scar 2 and 5 weeks after stroke, and found that scar diameter decreased over time without detectable differences between vehicle and pregabalin (Fig. 5F and G).

**Figure 6:**
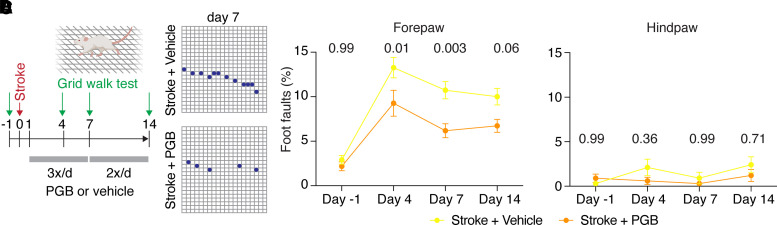
**Pregabalin improves skilled forelimb function after stroke**. (**A** and **B**) Experimental timeline and representative examples. (**C** and **D**) Foot fault frequency assessed for forelimb and hindlimb in mice treated with PGB after stroke compared with vehicle-treated mice after stroke (*n* = 11 mice per group; repeated-measures two-way ANOVA followed by Bonferroni’s multiple comparisons test).

Finally, we investigated the effect of pregabalin on motor outcome after stroke. To this end, we tested skilled forelimb function by quantifying foot faults of mice placed on an elevated grid (Fig. 6A). The test was performed 1 day before stroke and repeated 4, 7 and 14 days after stroke. As expected, the number of foot faults in vehicle-treated mice more than quadrupled 4 days after stroke compared with before stroke and remained high throughout the test period (compared with pre-stroke: Day 4, 456%; Day 7, 369%; Day 14, 344%; Fig. 6B and C). Remarkably, however, these motor deficits were strongly ameliorated by pregabalin at all time-points after stroke (compared with vehicle; Day 4, 70%; Day 7, 56%; Day 14, 67%; Fig. 3E and F; no difference between both groups was detectable 1 day before stroke; Fig. 6B and C). We did not test the effects of pregabalin beyond 14 days, as motor function returned to baseline in many vehicle-treated mice after 4–6 weeks, as has been reported for this stroke model.^17^ Consistent with the fact that the cortical representation of hindpaw function was outside of the stroke area, no differences were observed in skilled contralateral hindpaw use (Fig. 6D).

## Discussion

We here found that astrocyte-derived thrombospondin-1 is upregulated in the astroglial peri-infarct scar, and that treatment with the thrombospondin-1 receptor antagonist pregabalin induces axon sprouting and functional recovery after stroke.

We believe that these data are translationally relevant for several reasons. First, drugs such as pregabalin that promote axonal regeneration after stroke may have the potential to greatly expand the therapeutic time window, which is currently limited to the first few hours after stroke. Second, pregabalin has already been approved for other clinical indications, potentially enabling an easier translation into clinical trials and adding to the repertoire of drug-repurposing candidates for this indication.^4,18^ Third, pregabalin has also been shown to have beneficial effects on axon regeneration in preclinical and clinical spinal cord injury trials,^9,19^ in which thrombospondin-1 is also highly expressed in the glial scar,^20^ indicating utilization beyond stroke therapy. Fourth, a basic concept of developmental neurobiology posits that axon growth and synapse formation are based on mutually exclusive molecular programmes, and that the establishment of synaptic connections comes at the expense of a cessation of axon growth.^9^ While we did not investigate the formation and modulation of ectopic synapses in the glial scar in the present study, our data further corroborate that this developmental switch is recapitulated during CNS injury,^21^ in that synaptogenic cues such as thrombospondin-1 in the peri-infarct zone cause neurons to adopt a growth-incompetent mode. While the magnitude and time course of the modulation of ectopic synaptogenesis by pregabalin or other substances within the glial scar remain to be investigated by future studies, our data that the density of dystrophic axon segments is decreased by pregabalin support this notion. In that regard, it is interesting to note that axonal microtubule stabilization using the US Food and Drug Administration-approved drug epothilone also increased axon sprouting and motor outcome in a stroke model,^4^ supporting the concept of axon regeneration as an important translational drug target.

Interestingly, whereas we observed that retrograde connections between premotor and motor cortex were enhanced by pregabalin, the picture was more complex for anterograde connections, where anterolateral projections were enhanced but anteromedial connections decreased and no effect was seen on posterolateral connections. The cellular basis of these differential effects on axon regeneration remains to be determined, but possible reasons may include either a spatially heterogenous expression of α2δ1/2 in outgrowing axons, which also is the case in the developing brain,^22^ or a higher expression of other growth-inhibitory factors in some parts of the glial scar that may not be overcome by pregabalin alone. An alternative explanation is that axonal pruning may be necessary in some regions for successful circuit integration. Interestingly, data from spinal cord injury and other stroke models suggest that such axonal pruning of cortical or subcortical connections may be essential for functional integration into damaged circuits.^23^ This is indirectly supported by our finding of a trend toward an increase in the density of synaptic punctae in remote cortical projection areas in the pregabalin group. Our and previous data^24^ that thrombospondin-1 expression is not elevated in these regions indicate that different synaptogenic mechanisms may prevail in these remote areas. The mechanisms underlying these differential effects and levels of anatomical integration remain to be addressed in the future. Of note, our data also show that the therapeutic effects of pregabalin began to emerge within the first week of treatment. Although axonal sprouting may already start within a few days after stroke,^25,26^ and this process may potentially be accelerated by pregabalin, future studies may address other beneficial effects of pregabalin than those investigated here. It will also have to be determined whether benefits of pregabalin persist in stroke models that induce more chronic deficits than in the present model, and what the optimal time window for therapeutic modulation of synaptogenic pathways should be. The latter point appears critical as many synaptogenic cues overlap with angiogenic signals,^27^ and as TSP1/2 knockout mice as a model for long-term/lifelong complete blockade show deficits in recovery after stroke.^28^ In addition, as recovery-enhancing drug treatments in the clinical scenario would be applied in parallel to motor rehabilitation efforts, it will be interesting to investigate whether these interventions may stabilize the benefit on motor outcome, similar to what has been reported for other growth-promoting strategies.^29^

In conclusion, targeted inhibition of these synaptogenic signals may promote axon regeneration, structural plasticity and functional outcome after stroke. Pharmacological targeting of these pathways with gabapentinoids may represent a promising therapeutic strategy after stroke.

## Supplementary Material

fcac170_Supplementary_Data

## Abbreviations

AAV =: adeno-associated virus
CTb =: cholera toxin B
GO =: gene ontology
MCAO =: middle cerebral artery occlusion
PMC =: premotor cortex
THBS1 =: thrombospondin-1
