# Supplementary material for: Pregabalin improves axon regeneration and motor outcome in a rodent stroke model
Source: Brain Commun. 2022 Jun 27;4(4):fcac170. doi: 10.1093/braincomms/fcac170 (PMC9443992; doi:10.1093/braincomms/fcac170)
Supplement: fcac170_Supplementary_Data [file fcac170_Supplementary_Data.pdf]

Supplementary Material

Pregabalin improves axon regeneration and motor outcome in a rodent stroke model

Christof Kugler, Nelli Blank, Hana Matuskova, Nicole Reichenbach, Tien-Chen Lin, Christian Thielscher, Frank Bradke, Gabor C. Petzold

Supplementary Figure

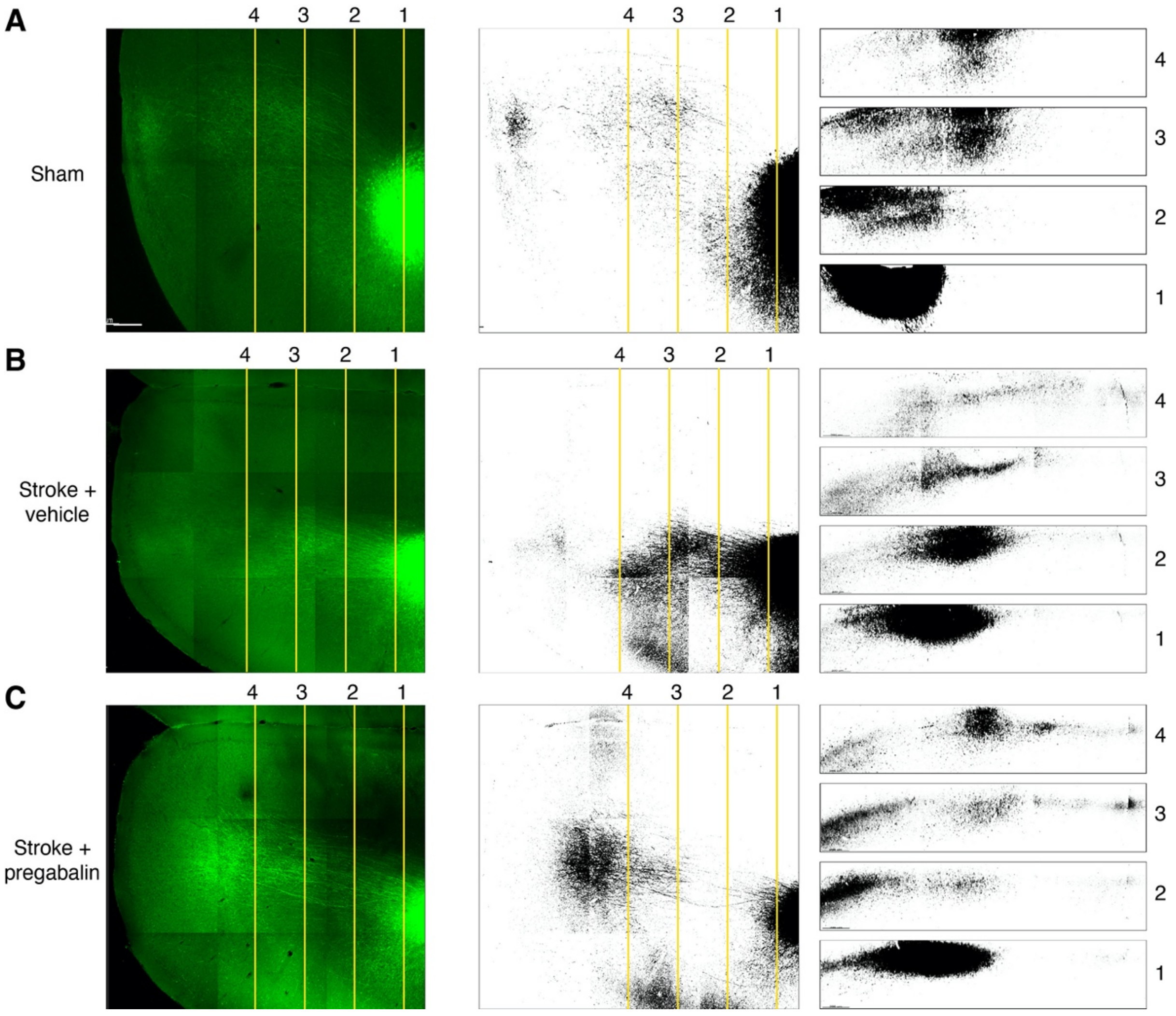

Supplementary Figure 1. Representative images of anterograde tracer (eGFP) in axonal projections. (**A-C**) Axial images stichted in Imaris (left; scale bar, 200 µm) were binarized (middle) and reconstructed coronally (right; intersectional distance, 350 µm).
